# Pharmacologic Effect of Miao Medicine* Illicium simonsii* Maxim. on Collagen-Induced Arthritis in Rats

**DOI:** 10.1155/2018/2398379

**Published:** 2018-10-11

**Authors:** Qing-zhong Zhang, Xian-yun Hu, Wen-qin Yao, Xiao-li Wan, Zi-cong Liang, Rong-min Dang, Zheng-min Yang, Ya-lan Xia

**Affiliations:** Qiannan Medicine College for Nationalities, Duyun, Guizhou 558000, China

## Abstract

**Objectives:**

To study the pharmacologic effect and mechanism of action of Miao medicine* Illicium simonsii* Maxim. (ISM) in treating rheumatoid arthritis.

**Methods:**

Sixty rats were randomly divided to six groups: normal control (normal), collagen-induced arthritis (CIA) model (model), CIA + tripterygium glycosides (TG), CIA + ISM high dose oral (ISM-H), CIA + ISM low-dose oral (ISM-L), and CIA + ISM topical application (ISM-T). The treatment doses were selected based on published reports and folk medicine practice. The outcome measurements included paw swelling, joint pathology, organ index, blood count, T helper 17 (Th17) cell count, and interleukin-6 (IL-6) level.

**Results:**

Compared to the CIA model group, all treatment groups showed a significant reduction in paw swelling, blood vessel pathology, Th17 cell count, and IL-6 levels (*p* < 0.05 or* p* < 0.01). All treatment groups showed alleviated foot swelling and lower total number of white blood cells, and these effects were observed earlier with oral ISM than topical ISM. The effect of ISM was weaker than that of TG. In addition, less organ damage was observed with topical ISM than oral ISM but better than TG.

**Conclusions:**

These results suggest that, by downregulating Th17 cells, ISM inhibits the production of Il-6, thereby alleviating the proliferation of endothelial and rheumatoid-like cells and leukocytosis in CIA rats, ultimately eliminating foot swelling.

## 1. Introduction

Rheumatoid arthritis (RA) is a chronic systemic disease characterized by inflammatory synovitis that mainly involves small joints of the hands and feet. RA is often accompanied by abnormalities involving extra-articular organs, joint deformity, and loss of function, thus negatively impacting physical and mental health, daily life, and economic status. Its etiology remains unclear. Approximately 0.3% and 0.5%–1% of the Chinese and global populations, respectively, are affected by RA [1–3]. Approximately 35% of patients with uncontrolled RA lose their ability to work within 10 years [4, 5].

Advances in nanotechnology, chemical synthesis, and enzymology [6–8] have extensively improve RA treatment. However, these have not been utilized in Chinese clinics due to the limiting factors such as inadequate medical facilities and their associated high cost. Compared to RA medicines such as tripterygium glycosides and methotrexate, there are several domestic drugs in China that are effective in treating RA [9, 10]. However, their application is limited because of their toxicity and side effects.

In Guizhou Miao medicine, people have traditionally employed* Illicium simonsii* Maxim. (ISM) to alleviate RA by either oral or topical application [11–15]. While some studies have conducted chemical and toxicological analyses of ISM [12, 13, 16, 17], no investigation on its therapeutic efficacy in RA has been conducted. The present study established a collagen-induced arthritis (CIA) rat model to determine the therapeutic efficacy of ISM on RA development and its effect on serum IL-6 levels, Th17 cell count [18], and synovial MMP-13 expression [19]. The results will help in establishing the medicinal applications of ISM.

## 2. Materials and Methods

### 2.1. Preparation of ISM

Dried ISM fruits were purchased from the Miao Region in Guizhou Province and authenticated by Prof. Xuejun Wei at the Qiannan Medical College for Nationalities (Duyun, Guizhou, China). ISM (5,000 g) was extracted thrice (30 min each time) using reflux extraction, and the resulting liquid was pooled. After rotary evaporation, 1,750 g of extract was obtained.

### 2.2. Animals and Reagents

Sixty specific pathogen-free male Sprague-Dawley (SD) rats (180 ± 20 g) were purchased from the Laboratory Animal Center of the Third Military Medical University, a center certified by the China Ministry of Public Health. The tripterygium glycosides were from the Deende Pharmaceutical, Inc. (Zhejiang, China). Complete Freund's adjuvant (CFA) was purchased from Sigma (Shanghai Greater China, China); IL-6, IL-17, and bovine collagen type II were purchased from Solarbio Science and Technology (Beijing, China), Wilking Biological Technology (Nanning, China), and Dingshengtai Biotechnology (Guizhou, China), respectively.

### 2.3. Animal Model Establishment and Drug Administration

The 60 SD male rats were randomly divided into six groups (10 rats/group), which included a normal control group and five CIA disease model groups. All experiments were conducted following the animal welfare and protection regulations. Type II bovine collagen was dissolved in acetic acid (0.05 mol/L) to make a 2 mg/mL solution, which was incubated with shaking at 4°C overnight. Equal volumes of type II bovine collagen solution and CFA were mixed to make an emulsion, of which 0.1 mL was injected to the right hind food pad of each rat to induce CIA. The CIA rats were divided into five treatment groups (10 rats/group): CIA model (model), CIA + tripterygium glycosides (TG), CIA + ISM high-dose oral (ISM-H), CIA + ISM low-dose oral (ISM-L), and CIA + ISM topical application (ISM-T). Approximately 10 days after CIA induction, the rats received their respective treatments. The rats in the normal control group (normal) were given saline via gavage, and those in the ISM-T group were treated externally using their right hind foot and ankle joint. The rats in the TG treatment group were given a daily gavage of tripterygium glycosides at a dose of 50 mg/kg body weight [20]. The rats in oral ISM high-dose (ISM-H) and low-dose (ISM-L) groups were given a daily gavage of 2.1 g and 0.7 g/kg body weight, respectively. These oral doses of ISM were selected based on folk medicine practice and reported pharmacotoxicology results [14]. The rats in the ISM-T group were given ISM daily at a dose of 3.5 g/kg body weight by local application to the right hind foot and ankle joints after shaving the treatment site to remove any hair.

### 2.4. Foot Swelling

At −1, 3, 7…… 27 days (once every four days) after treatment, swelling of the right hind foot joints was measured with a caliper.

### 2.5. Organ Indices

The day after the last drug administration, the rats were anesthetized by peritoneal injection of 10% chloral hydrate (0.1 mL/100 g kg body weight) [19] and then euthanized by cervical dislocation. The spleen, kidneys, liver, lungs, and thymus were collected and weighed for organ index calculation.

### 2.6. Serum IL-6 Analysis by ELISA

Blood (2.5 mL) was collected after the rats were euthanized. Blood was left to stand for 1 h and then centrifuged at 2,000 rpm for 15 min to obtain the serum. Serum IL-6 was quantified using an ELISA kit following the manufacturer's instructions.

### 2.7. Measurement of Th17 Cells by Flow Cytometry

After the rats were euthanized as earlier described, 250 *μ*L of whole blood was collected and diluted with RPMI 1640 medium at a ratio of 1 : 1, resulting in a total volume of 500 *μ*L. Approximately 2 *μ*L of a stimulator/blocker solution was added to the diluted blood and then incubated at 37°C in 5% CO_2_ for 4 h. After centrifugation and removal of the supernatant, red blood cell lysis buffer was added (2 mL/100 *μ*L whole blood), and the mixture was incubated in the dark for 10 min. Then, the samples were centrifuged to remove the supernatant, and then 5 *μ*L of FITC-labeled anti-CD4 was added and thoroughly mixed. After incubation in the dark at 2–8°C for 15 min, 2 mL of a staining buffer was added to resuspend the cells, followed by centrifugation at 1,200 rpm for 5 min. After removing the supernatant, 250 *μ*L of a Fix/Perm buffer was added, mixed, and then incubated in the dark at 2–8°C for 20 min, at which an additional 1 mL of 1× Perm/Wash buffer was added and mixed. After centrifugation, part of the supernatant was removed, leaving 250 *μ*L of liquid, upon which 1 mL 1× Perm/Wash buffer was added to resuspend the cells. After another centrifugation at 1,200 rpm for 5 min, the supernatant was removed and 80 *μ*L to 100 *μ*L of the stain buffer was added to resuspend cells, to which 5 *μ*L of PE-labeled IL-17A antibody was added, mixed, and incubated in the dark for 30 min. Next, 2 mL of 1× Perm/Wash buffer was added to resuspend the cells, which were again centrifuged at 1,200 rpm for 5 min, and then the supernatant was removed. Finally, 500 *μ*L of the stain buffer was added to resuspend the cells, which were then passed through a 300-*μ*m filter, followed by flow cytometry analysis.

### 2.8. HE Staining of Foot Joints

After the rats were euthanized, the foot joints were collected and fixed with 4% paraformaldehyde, followed by a series of processes, including fixation, decalcification, deacidification, dehydration, embedding, and sectioning. The tissue sections were examined under a microscope.

### 2.9. Blood Tests

After the rats were euthanized, 3 mL whole blood was collected for routine blood testing.

### 2.10. Statistical Analysis

The measurement data were expressed as mean ± standard deviation ($\overset{-}{X}\;\;$±  SD). The differences among groups were analyzed by one-way ANOVA, and the LSD test was used for pairwise comparisons.* p *< 0.05 was considered statistically significant.

## 3. Results

### 3.1. Foot Swelling

Table 1 shows that, compared to the CIA model group, the normal group (from day 3), the TG, ISM-H, and ISM-L oral groups (all from day 19), and ISM-T group (from day 27) all exhibited significant attenuation of foot swelling (*p* < 0.05 or* p* < 0.01). No significant difference between each administration group and TG group (except on the 23rd day), as well as between the ISM-T group and the ISM-oral group (*p* > 0.05), was observed.

### 3.2. Organ Indices

Table 2 shows that, compared to the model group, the normal group, the TG group, and the ISM-T group had significantly lower lung index (*p* < 0.05 or* p* < 0.01), and the spleen and thymus indexes were also significantly lower in the TG group (*p* < 0.05). Compared to the TG group, the ISM-H and ISM-L groups had higher spleen index (*p* < 0.05), the ISM-H group and ISM-T group had higher liver index (*p* < 0.01), and the ISM-H group had higher lung index (*p* < 0.05). Compared to the ISM-T group, the ISM-H group had higher spleen, liver, and lung indexes (*p* < 0.05).

### 3.3. Serum IL-6 Levels

**Figure 1:**
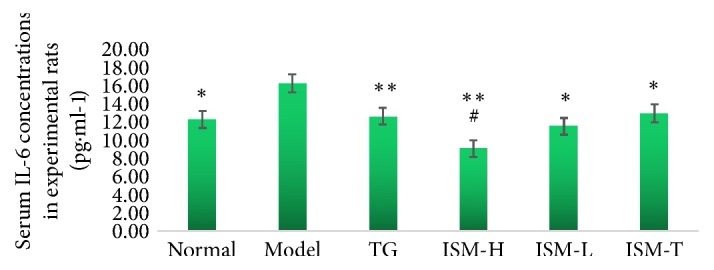
Serum IL-6 concentrations in experimental rats. Note: ^∗^*p* < 0.05, ^∗∗^*p* < 0.01, vs model group; ^#^*p* < 0.05, vs TG group.

Figure 1 shows that, compared to the CIA model group, the normal group, the TG group, the ISM-H and ISM-L groups, and the ISM-T group had significantly lower serum IL-6 levels (*p* < 0.05 or* p* < 0.01). This reduction was greater in the ISM-H group compared to the TG group (*p* < 0.05).

### 3.4. Th17 Cells

**Figure 2:**
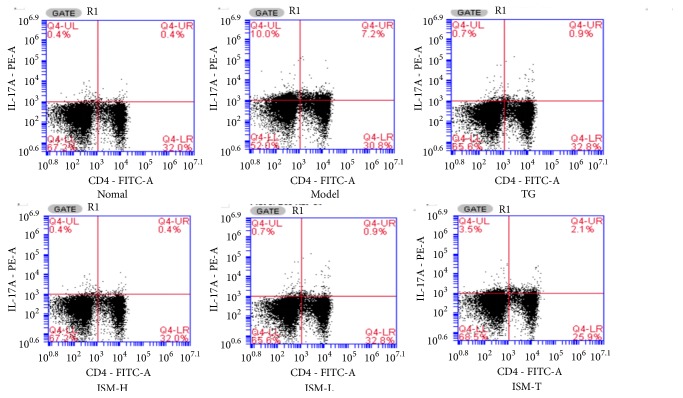
Flow cytometry analysis of Th17 cells in the experimental rats.

**Figure 3:**
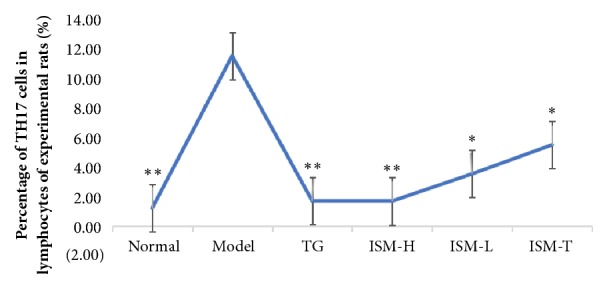
Percentage of Th17 cells in the lymphocytes of the experimental rats. Note: ^∗^*p* < 0.05, ^∗∗^*p* < 0.01, vs model group.

Figure 2 shows the Th17 populations in different groups as determined by flow cytometry analysis. Compared to the CIA model group, the percentage of Th17 cells in the total number of lymphocytes was significantly lower in all the other groups (*p* < 0.05 or* p* < 0.01). No significant difference between the ISM treatment groups and the TG group, or between the ISM oral groups and the topical group (*p* > 0.05), was observed (Figure 3).

### 3.5. Pathology (HE Staining)

**Figure 4:**
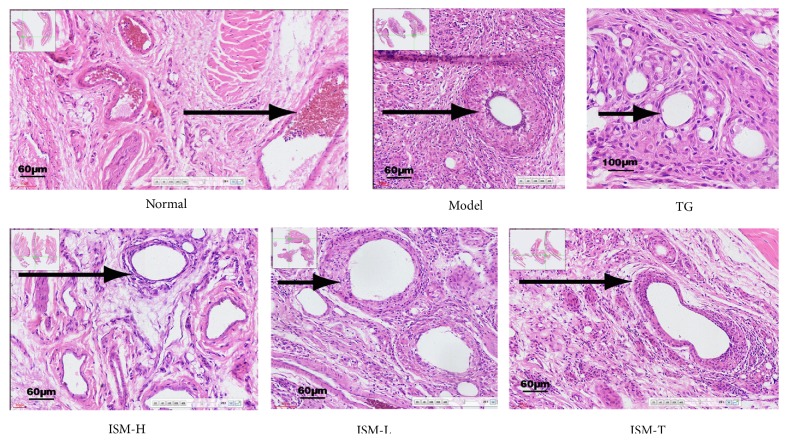
Pathology of rat foot and ankle joints (HE staining, 20×).

Figure 4 shows the pathological sections of the joints. The blood vessels in the feet of the CIA model rats showed hyperplasia in rheumatoid-like cells, endothelial cells, epithelial cells, and fibrous tissues (Figure 4). Based on the joint pathological scoring method [21], the rats in the ISM-H and ISM-L groups, ISM-T groups, and the TG group all showed significant improvement in reducing hyperplasia in blood vessel fibrous tissues, endothelial cells, and rheumatoid-like cells (*p* < 0.05 or* p* < 0.01). The effect on the ISM-H, ISM-L, and ISM-T groups in reducing hyperplasia of rheumatoid-like cells, endothelial cells, epithelial cells, and fibrous tissues was less significant than that in the TG group (*p* < 0.05 or* p* < 0.01). Compared to the ISM-T group, the ISM-H group showed more extensive improvement in reducing endothelial cell proliferation (*p* < 0.05), and the ISM-H and ISM-L groups showed more significant improvement in reducing the number of rheumatoid-like cells (*p* < 0.05 or* p* < 0.01) (Table 3).

### 3.6. Blood Test

Compared to the CIA model group, the normal, TG, ISM-H, and ISM-L groups had significantly fewer total white blood cells (WBCs) (*p* < 0.05 or* p* < 0.01). The normal, TG, ISM-H, and ISM-L groups had significantly fewer monocytes (*p* < 0.05 or* p* < 0.01). The ISM-H and ISM-T groups had more eosinophils (*p* < 0.05 or* p* < 0.01). The TG group had fewer total platelet counts, whereas the ISM-T group had more (*p* < 0.05). Compared to the TG group, the ISM-T group had more neutrophils (*p* < 0.05), the ISM-H, ISM-L, and ISM-T groups had more monocytes (*p* < 0.05 or* p* < 0.01), and the ISM-H and ISM-T groups had more eosinophils (*p* < 0.05 or* p* < 0.01) and total platelet counts (*p* < 0.01) (Table 4).

## 4. Discussion

### 4.1. Pharmacological Efficacy, Dose, and Effect of ISM on Organs

Comparison of foot swelling among different treatment groups (Table 1) showed that the well-recognized anti-RA drug tripterygium glycoside and both oral and topical ISM administration could significantly improve foot swelling, and this effect was not significantly different among treatments, except that the effects of ISM were observed later when topically administered. These results indicate that ISM is effective in improving foot swelling in CIA rats, and oral administration is superior to topical application. Furthermore, this effect of ISM is similar to the well-recognized anti-RA drug tripterygium glycoside.

Table 2 shows that the spleen, liver, and lung indices were higher with topical ISM than oral, and the spleen, liver, and lung indices downward effects of ISM, regardless of administration, were significantly better than that using tripterygium glycosides. These results showed that ISM is more effective in the treatment of CIA in rats than tripterygium glycosides.

Pathological scores of the joint sections (Table 3) demonstrated that ISM application significantly alleviated toe vascular fibrosis and improved endothelial cell proliferation and the number of rheumatic-like cells in the CIA rats. ISM oral administration has significantly stronger effectiveness than topical use, but weaker than tripterygium glycosides. However, ISM has fewer side effects than tripterygium glycosides.

Table 4 shows that both ISM oral and topical administration can cut down the total number of leukocytes and monocytes in rats, but the reduction was more pronounced with oral ISM, and the percentage of eosinophils and the total number of platelets in the topical ISM group significantly increased. The effect of the oral ISM administration is better than topical administration and did not significantly differ from the well-recognized anti-RA drug tripterygium glycoside.

### 4.2. Mechanistic Study of the Anti-RA Effect of ISM

IL-6 is an important cytokine derived from monocytes/macrophages, and its interaction with several other cytokines is essential to the induction of RA [18, 22, 23]. Th17 cells produce IL-17, which is an inflammatory cytokine that has potent proinflammatory activity and works with other cytokines to amplify inflammatory responses [24, 25]. IL-17 can stimulate the production of IL-6 and monocyte chemoattractant protein-1 (MCP-1) by synovial cells, fibroblasts, and endothelial cells [25, 26]. The present study observed that ISM, through both oral and topical administration, significantly reduced IL-6 levels (Figure 1) and the number of Th17 cells (Figure 3). Assessment of foot swelling, toe histopathological sections, and blood routine studies indicate that the IL-6 levels and the number of Th17 cells are positively correlated with foot swelling, cell proliferation, and the increase of leukocyte count, which agrees with the results of a previous study [27]. Therefore, by downregulating Th17 cells, ISM may inhibit the production of a variety of cytokines such as Il-6 [25, 26], reduce endothelial cells to express adhesion molecules, and consequently decrease monocyte migration to the inflammation sites [28, 29], and then inhibit multiple cellular responses [cell multiplication, cell differentiation, enhancement of immune response, and angiogenesis [18]] to alleviate the proliferation of endothelial cells and rheumatoid-like cells and leukocytosis in CIA rats. In addition, ISM is superior to tripterygium glycosides for the treatment of CIA.

## 5. Conclusions

ISM is a Miao medicine that is commonly used for the treatment of RA. ISM is widely distributed in China, has few side effects, and can be used both orally and topically. By downregulating Th17 cells, ISM inhibits the production of Il-6 and reduces the expression of adhesion molecules in endothelial cells, consequently decreasing monocyte migration to the inflammation sites, alleviating the proliferation of endothelial and rheumatoid-like cells and leukocytosis, ultimately eliminating foot swelling in CIA rats.

## Figures and Tables

**Table 1:** Rat foot swelling (mm).

Time (d)	Normal	Model	TG	ISM-H	ISM-L	ISM-T
−1	4.73 ± 1.08	l4.79 ± 0.43	4.81 ± 0.79	4.74 ± 0.65	4.68 ± 0.51	4.85 ± 0.64
3	5.02 ± 1.26^∗∗^	7.47 ± 2.45^∗∗^	7.58 ± 2.56^∗∗^	7.63 ± 2.61^∗∗^	7.50 ± 2.48^∗∗^	7.66 ± 2.65^∗∗^
7	5.22 ± 1.18^∗∗^	7.50 ± 0.08	7.69 ± 1.38	7.50 ± 1.34	7.45 ± 0.68	7.74 ± 1.07
11	5.52 ± 0.89^∗∗^	7.88 ± 1.53	7.76 ± 1.05	7.81 ± 0.98	8.01 ± 1.08	7.91 ± 1.28
15	5.78 ± 0.58^∗∗^	7.82 ± 1.19	7.69 ± 1.09	7.70 ± 0.68	7.75 ± 1.05	7.69 ± 1.08
19	6.03 ± 0.74^∗∗^	7.65 ± 1.08	6.89 ± 0.98	7.03 ± 0.91^∗^	7.09 ± 0.87^∗^	7.50 ± 1.02
23	6.15 ± 1.02^∗∗^	7.41 ± 1.01	6.36 ± 0.75^∗∗^	6.81 ± 0.78^∗∗#^	7.06 ± 1.01^∗##^	7.20 ± 0.88^#^
27	6.28 ± 1.19^∗∗^	7.15 ± 1.57	6.21 ± 0.88^∗∗^	6.55 ± 0.58^∗∗^	6.35 ± 0.73^∗∗^	6.66 ± 0.62^∗^

Note: ^*∗*^*p* < 0.05, ^*∗∗*^*p* < 0.01, vs model group; ^#^*p* < 0.05, ^##^*p* < 0.01, vs TG group.

**Table 2:** Rat organ index.

Group	Spleen	Kidney	Liver	Lung	Thymus
Normal	0.19 ± 0.08	0.73 ± 0.01	3.71 ± 0.31	0.51 ± 0.12^∗^	0.12 ± 0.05
Model	0.25 ± 0.03	0.73 ± 0.01	4.14 ± 0.81	0.56±0.04	0.16 ± 0.02
TG	0.18 ± 0.01^∗^	0.73 ± 0.02	3.73 ± 0.39	0.46±0.07^∗∗^	0.12 ± 0.08^∗^
ISM-H	0.23 ± 0.02^#@^	0.74 ± 0.03	4.49 ± 0.39^##@^	0.52±0.06^#@^	0.14 ± 0.01
ISM-L	0.23 ± 0.06^##^	0.71 ± 0.03	4.04 ± 0.48	0.50±0.06	0.14 ± 0.01
ISM-T	0.20 ± 0.02	0.73 ± 0.07	3.93 ± 0.77^##^	0.49±0.09^∗∗^	0.13 ± 0.04

Note: ^∗^*p* < 0.05, ^∗∗^*p* < 0.01, *vs*. model group; ^#^*p* < 0.05, ^##^*p* < 0.01, *vs.* TG group; ^@^*p* < 0.05, *vs*. ISM-T group.

**Table 3:** Rat foot joint pathological scores.

Group	Blood vessel fibrosis	Endothelial proliferation	Rheumatoid-like cells
Normal	0	0	0
Model	1.66 ± 0.09	2.45 ± 0.05	1.14 ± 0.03
TG	0.49 ± 0.07^∗∗^	0.80 ± 0.04^∗∗^	0.22 ± 0.03^∗∗^
ISM-H	1.18 ± 0.02^∗∗##^	2.13 ± 0.05^∗∗##@^	0.70 ± 0.07^∗∗##@@^
ISM-L	1.38 ± 0.17^∗∗##^	2.22 ± 0.02^∗∗##^	0.90 ± 0.04^∗∗##@^
ISM-T	1.41 ± 0.09^∗∗##^	2.34 ± 0.01^∗∗##^	1.10 ± 0.07^∗##^

Note: ^∗^*p* < 0.05, ^∗∗^*p* < 0.01, vs model group; ^##^*p* < 0.01, vs TG group; ^@^*p* < 0.05, ^@@^*p* < 0.01, vs ISM-T group.

**Table 4:** Rat blood test.

Group	Total white blood cell count	Percentage of neutrophils	Percentage of lymphocytes	Percentage of monocytes	Percentage of eosinophils	Total platelet count
Normal	3.25 ± 0.37^∗∗^	0.20 ± 0.02	0.76 ± 0.11	0.06 ± 0.01^∗∗^	0.03 ± 0.01	297.00 ± 0.98
Model	4.82 ± 0.27	0.34 ± 0.06	0.58 ± 0.09	0.21 ± 0.04	0.06 ± 0.02	266.50 ± 1.38
TG	2.89 ± 0.67^∗^	0.18 ± 0.05	0.74 ± 0.10	0.09 ± 0.05^∗^	0.06 ± 0.01	186.50 ± 1.35^∗^
ISM-H	3.50 ± 0.38^∗^	0.14 ± 0.02^@^	0.72 ± 0.08	0.15 ± 0.02^∗#^	0.09 ± 0.03^∗#@^	424.50 ± 2.30^#^
ISM-L	3.30 ± 0.56^∗^	0.23 ± 0.03	0.70 ± 0.06	0.12 ± 0.03^∗#@@^	0.06 ± 0.01^@@^	243.00 ± 1.76^#@@^
ISM-T	3.94 ± 0.36	0.28 ± 0.02^#^	0.65 ± 0.07	0.21 ± 0.02^##^	0.13 ± 0.01^∗∗##^	363.88 ± 2.58^∗#^

Note: ^∗^*p* < 0.05, ^∗∗^*p* < 0.01, vs model group; ^#^*p* < 0.05, ^##^*p* < 0.01, *vs*. TG group; ^@^*p* < 0.05, ^@@^*p* < 0.01, *vs*. ISM-T group.

## Data Availability

The data used to support the findings of this study are available from the corresponding author upon request.
